# The Health-e Babies App for antenatal education: Feasibility for socially disadvantaged women

**DOI:** 10.1371/journal.pone.0194337

**Published:** 2018-05-16

**Authors:** Julia A. Dalton, Dianne Rodger, Michael Wilmore, Sal Humphreys, Andrew Skuse, Claire T. Roberts, Vicki L. Clifton

**Affiliations:** 1 Robinson Research Institute, University of Adelaide, Adelaide, South Australia, Australia; 2 Department of Anthropology and Development Studies, School of Social Sciences, University of Adelaide, Adelaide, South Australia, Australia; 3 Department of Media Studies, School of Social Sciences, University of Adelaide, Adelaide, South Australia, Australia; 4 Faculty of Health, Arts and Design, Swinburne University, Hawthorn, Victoria, Australia; Karolinska Institutet, SWEDEN

## Abstract

**Background:**

The use of mobile technology such as phone applications (apps) has been proposed as an efficient means of providing health and clinical information in a variety of healthcare settings. We developed the Health-e Babies app as an Android smart phone application for pregnant women attending a tertiary hospital in a low socio-economic community, with the objective of providing health information about early pregnancy that would increase maternal confidence and reduce anxiety. Based on our earlier research, this form of health communication was viewed as a preferred source of information for women of reproductive age. However, the pilot study had a poor participation rate with 76% (n = 94) not completing the study requirements. These initial findings raised some very important issues in relation to the difficulties of engaging women with a pregnancy app. This paper analyses the characteristics of the participants who did not complete the study requirements in an attempt to identify potential barriers associated with the implementation of a pregnancy app.

**Methods:**

This retrospective review of quantitative and qualitative data collected at the commencement of the Health-e Babies App trial, related to the participant’s communication technology use, confidence in knowing where to seek help and mental health status, maternal-fetal attachment and parenting confidence. Engagement and use of the Health-e Babies App was measured by the completion of a questionnaire about the app and downloaded data from participant’s phones. Mental health status, confidence and self-efficacy were measured by questionnaires.

**Results:**

All women were similar in terms of age, race, marital status and level of education. Of the 94 women (76%) who did not complete the trial, they were significantly more anxious as indicated by State Trait Anxiety Inventory (p = 0.001 Student T-test) and more likely to be unemployed (50% vs 31%, p = 0.012 Student T-Test).

**Conclusion:**

This study provides important information about the challenges associated with the implementation of a pregnancy app in a socially disadvantaged community. The data suggests that factors including social and mental health issues, financial constraints and technological ability can affect women’s engagement with a mobile phone app.

## Introduction

In a world of increasing use of information and communication technology, the internet and pregnancy mobile applications (apps) are very popular media for pregnant women seeking pregnancy-related health information [1, 2]. Increased use of these media may potentially improve women’s understanding and knowledge of personal health and healthcare options and enhance their ability to make the most appropriate choices for pregnancy. However, barriers still prevent some women from engaging with antenatal educational materials. This is a concern for health professionals given that health literacy during pregnancy is vitally important for maternal and fetal health and wellbeing [3–5]. There is a direct correlation between low health literacy and poor health outcomes [5] and between low socio-economic status (SES) and poor health outcomes [6–8], so it is important to promote health education.

The popularity of mobile pregnancy apps has increased irrespective of socio-economic status [1, 9]. Whilst there are strong advocates for the use of mobile technology in childbirth education [10], few pregnancy apps have undergone scrutiny to evaluate the accuracy of the information they provide and their alignment with current obstetric guidelines [11]. Daniels and Wedler (10) claim that pregnant women are technologically literate and able to navigate the internet and mobile apps for self-education, but fail to offer suggestions as to how to best engage women, particularly if they have literacy issues or lack interest in self-learning.

A randomised controlled trial that consisted of the use of a pregnancy app compared with a paper-based tool, showed that there was increased engagement and active learning by app users [12]. However this study was limited to women with tertiary education and low risk pregnancies. Women living in low socio-economic circumstances and with limited education were not included. It may be that engagement with mobile apps may vary between socio-economic status (SES) groups but this is yet to be determined.

Our research focussed on health outcomes in pregnant women from a socially disadvantaged population [13–16]. The level of social deprivation in this pregnant population is significant. For example 36% of pregnant women from this population have reported they were abused as a child, 35% have suffered major life stressors and 30% of these pregnant women were diagnosed with depression during their antenatal care [16]. Population based data reported that 40% of the inhabitants in this community do not finish high school beyond year 10, greater than 22% of the population are unemployed, 27% are housed by the government and 22% of families have a sole female parent [17]. Within this population we found that, despite social disadvantage, all women had a mobile phone and wanted pregnancy related health information that was relevant to their specific needs, was easily understood and accessible at any time [1]. Based on this evidence, we hypothesised that a phone app targeted specifically at a low level of literacy and designed to provide hospital specific information, as well as pregnancy information, would be an effective tool to increase the population’s ability to seek information, gain interest in self-learning, increase confidence and reduce anxiety around pregnancy related issues. Consequently, we created an Android mobile app called the ‘Health-e Babies App’ which was specifically for pregnant women attending a tertiary hospital that was the main provider of antenatal and obstetric care for this population.

**Fig 1:**
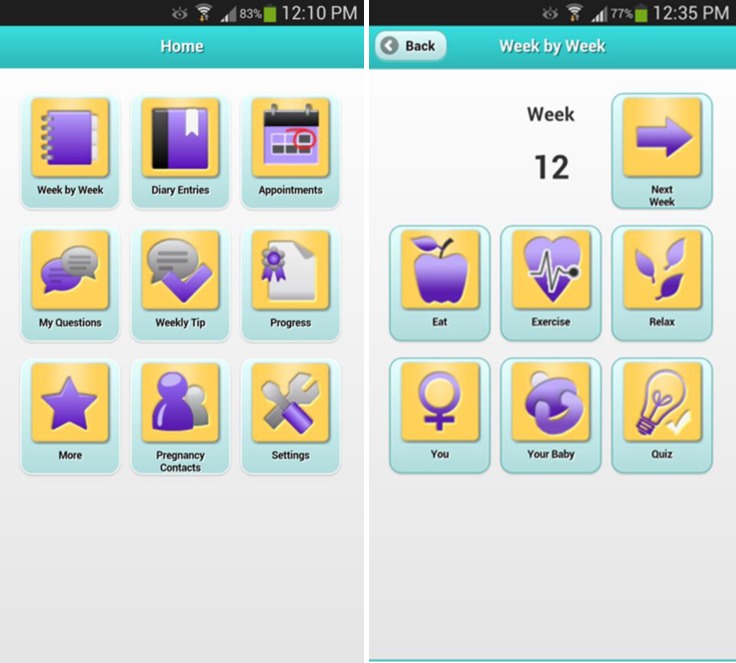
Health-e Babies App screen shot.

The Health-e Babies App provided information regarding fetal development, maternal physical changes, explanations of the tests and procedures they may undergo and problems they may experience during their pregnancy with advice on what to do and where to seek help if needed. It promoted healthy eating, exercise and relaxation during pregnancy with scientifically based information with particular emphasis on relieving anxiety and depression. It was also aimed to enable easy access to hospital, community health services, support groups and research-based websites, should the participant require further in-depth information or assistance. (Fig 1). Notifications were also embedded into the app to remind participants of appointments and also if they had not used the app for a few weeks, it would suggest that they engage with it.

The initial development of the app included consumer participation with focus groups of pregnant women at 3 major stages of the app development to ensure their needs were met in terms of design, literacy, content and useability. The next stage of the research was to test the app in a cohort of pregnant women and this paper will report on the findings from this stage of the project. In particular we will focus on the characteristics of women who used the phone app relative to those who did not.

Participants in the focus groups were from the same socio-economic region and attending the same hospital as the participants who participated in the app trial, however the focus group participants were not included in the trial.

## Method

### Participants

The study was approved by the hospital’s Human Research Ethics Committee (TQEH/LMH/MH), application number 2011026. Pregnant women attending their first antenatal appointment at the trial hospital were invited to participate in the study. Eligibility for recruitment included women aged 18 years or older, pregnant between 10 and 14 weeks gestation at the time of recruitment and those who had an Android mobile phone or tablet. Women could be nulliparous or multiparous. Exclusion criteria included greater than 14 weeks gestation because it was considered that participants may potentially be limited by time because the app focused on a 10 week, second trimester period. As this study was a pilot for the possible development of a full pregnancy app, the research team decided not to conduct a randomised control trial at this stage of the project.

Immediately after attaining an informed signed consent, participants were sent a text message to their mobile phones or an email that contained a link to enable the Health-e Babies App to be downloaded. Verbal and written information about how to download the app was given at the time of recruitment. Participants were only given information on how to download the app and the initial set-up data entry requirements, such as name, date of birth, due date for the baby’s birth, their own local general practitioner’s name and phone number and future appointment dates. Participants were expected to download and navigate the app without further encouragement by the researcher to use any particular aspect of the app. The purpose of this approach forms part of the initial assessment of the app in relation to determining how easy it was for participants to use the app and how many women were motivated to explore and learn from it. Most participants did not download the app in the presence of the researcher but did so after the appointment.

Qualitative and quantitative methodologies were used for the evaluation of the Health-e Babies App. Participants were given questionnaires at the time of commencement and completion of the trial. Questionnaires given at recruitment consisted of demographic information and their use of the internet and other mobile pregnancy applications (S1 File) This was to identify the socio-economic status of the trial participants and their level of information and communication technology (ICT) use for pregnancy-related health information. This survey included a 7 point self-efficacy scale (0–6), to determine their level of confidence in knowing where to get health information and/or help if needed.

The evaluation of depression, anxiety, maternal-fetal attachment and parenting confidence was focussed on assessing any effects the app may have had during the course of pregnancy. Questionnaires at recruitment consisted of depression and anxiety evaluation; Edinburgh Postnatal Depression Score- EPDS, Antenatal Risk Questionnaire- ANRQ, State Trait Anxiety Inventory-STAI, Generalised Anxiety Disorder–GAD-7, Maternal Antenatal Attachment Score- MAAS for maternal and fetal attachment [18] and Parenting Sense of Competence PSoC [19, 20] (S1 File, S2 File, S3 File) These questionnaires were included in the study to assist in identifying the participants’ mental health status before and after the trial period because maternal mental health status can significantly affect confidence levels [21]. Each of these evaluation tools has been validated by previous research [22–26]. ANRQ reflects life and social experience and its effect on depression and anxiety [22]. The EDPS examines the level of anxiety, depression and ability to cope over the last 7 days [23, 27]. The GAD-7 and STAI assesses current anxiety levels. The MAAS is used to evaluate maternal and fetal attachment, recognizing the factors of tolerance, acceptance, pleasure of interaction and competence of parenting [28]. The Maternal Antenatal Attachment Survey indicated that the higher the score, the closer the maternal-fetal attachment. The PSoC is a 17 point assessment of self-confidence in competence of parenting that comprises of sub sections relating to satisfaction, efficacy, interest and control [19, 20, 26]. Scoring for the Parenting Sense of Competence questionnaire indicated that the lower the score, the more confident the participant felt at parenting.

Questionnaires related to app usage and opinions about the Health-e Babies App were administered at the end of the trial. The self-efficacy scale, level of anxiety, maternal and fetal attachment and confidence in parenting questionnaires were also administered at the end of the trial (S4 File, S3 File).

### Statistical analysis

Participants who completed the study requirements and used the Health-e Babies App were named the ‘App users’. Those who did not complete the study requirements were named the ‘Non-app users’, however, it could not be determined whether or not these women used the Health-e Babies App. The inability to remotely monitor participant’s app usage is examined in the limitations section of this paper. The differences between App users and Non-app users were examined using unpaired t-tests for parametric continuous variables, and Chi-Square test for categorical variables, following stratification compliance. All reported *P* values were two-tailed, and a *P* value of <0.05 was considered to be statistically significant. All statistical procedures were carried out using SPSS version 24 (SPSS Inc., Chicago, IL, USA).

## Results

**Fig 2:**
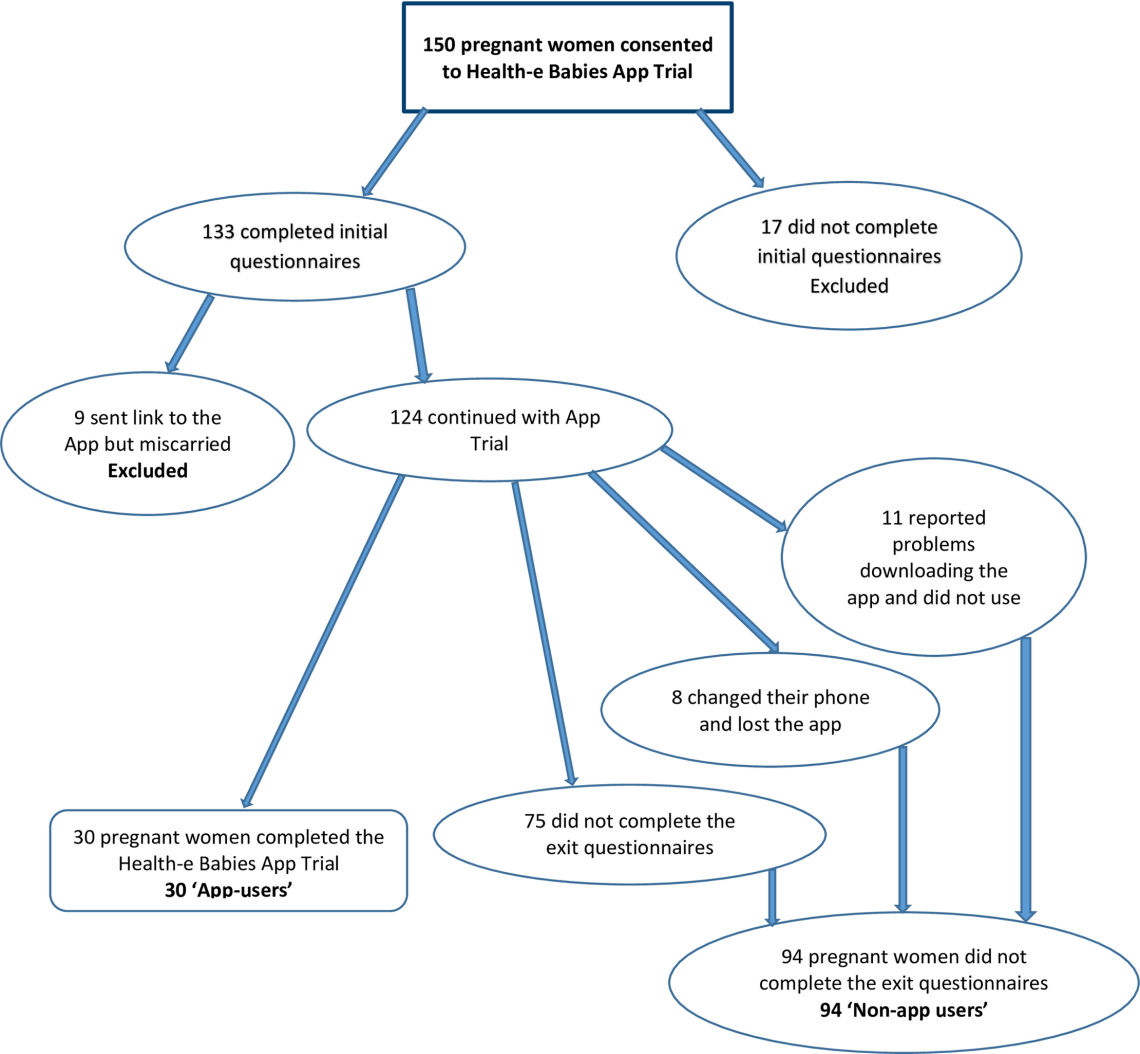
Recruitment flowchart.

One hundred and fifty pregnant women consented to participate in the study. Nine women (6%) suffered a miscarriage and 11% (n = 17) did not complete all of the initial questionnaires resulting in exclusion from the study. This left a cohort of 124 women who went forward in the study. Difficulties arose with 9% (n = 11) of women failing to report problems in downloading the app, despite being given contact phone numbers for technological support. Few women reported any problems prior to the trial period ending. In addition to this, when contacted at the end of the trial period, several women reported that they had changed mobile phones or lost their phones thereby losing access to the app (6%, n = 8). Sixty percent (n = 75) of participants failed to complete the exit questionnaires, therefore 30 women (24%) from the 124 participants completed the trial (Fig 2).

### Demographics

Of the thirty App user participants, the average age was 25 years (range 19–41 years). Most women identified themselves as Caucasian Australian (83%, n = 24) and 55% (n = 16) were nulliparous. Non-app users were predominantly nulliparous women (n = 85, 89%). This was due to the researcher’s access to more nulliparous women during the recruitment period and not associated with a more profound interest in the study than multiparous women.

There was no significant difference in relation to parity within the App-user group (nulliparous n = 17 and multiparous n = 13 (Student t-test, p = 0.856).

As indicated in Table 1, comparing the demographics of App user participants with the Non- app user participants, there was no significant difference between the two cohorts in relation to age, race, marital status, education and the possession of a government Healthcare Card (reflecting low income). However there were significant differences in relation to employment status (Student t-test, p = 0.012) indicating a higher rate of unemployment in the Non-app user group.

**Table 1:** Demographics.

	APP USER	NON-APP USER	P value
**AGE**			.289
Mean	26.9667	25.7340	
N	30	94	
Standard Error	1.03888	0.48594	
Range	19–41 yrs	18–41 yrs	
**PARITY**			.002*
Nulliparous (n, %)	17 (57%)	84 (89%)	
Multiparous (n, %)	13 (43%)	10 (11%)	
**MARITAL STATUS**			.183
Never Married (n, %)	3 (10%)	23 (24%)	
Married/Defacto (n, %)	27 (90%)	67 (72%)	
Separated (n, %)	0 (0%)	2 (2%)	
Divorced (n, %)	0 (0%)	1 (1%)	
**EDUCATION**			.639
Year 9 (n, %)	0 (0%)	3 (3%)	
Year 10 (n, %)	2 (7%)	20 (21%)	
Year 11 (n, %)	7 (23%)	15 (16%)	
Year 12 (n, %)	12 (40%)	24 (25%)	
Certificate (n, %)	4 (13%)	20 (21%)	
University degree (n, %)	5 (17%)	12 (13%)	
**EMPLOYMENT STATUS**			**.012** *
Unemployed (n, %)	1 (3%)	15 (16%)	
Home duties (n, %)	6 (20%)	22 (23%)	
Student (n, %)	0 (0%)	10 (11%)	
Employed (n, %)	23 (77%)	47 (50%)	
**HEALTHCARE CARD**			.060
Yes (n, %)	8 (28%)	42 (44%)	
No (n, %)	22 (72%)	52 (56%)	

* = P = <0.05 Significant difference

### Information and communication technology (ICT) use prior to recruitment

**Fig 3:**
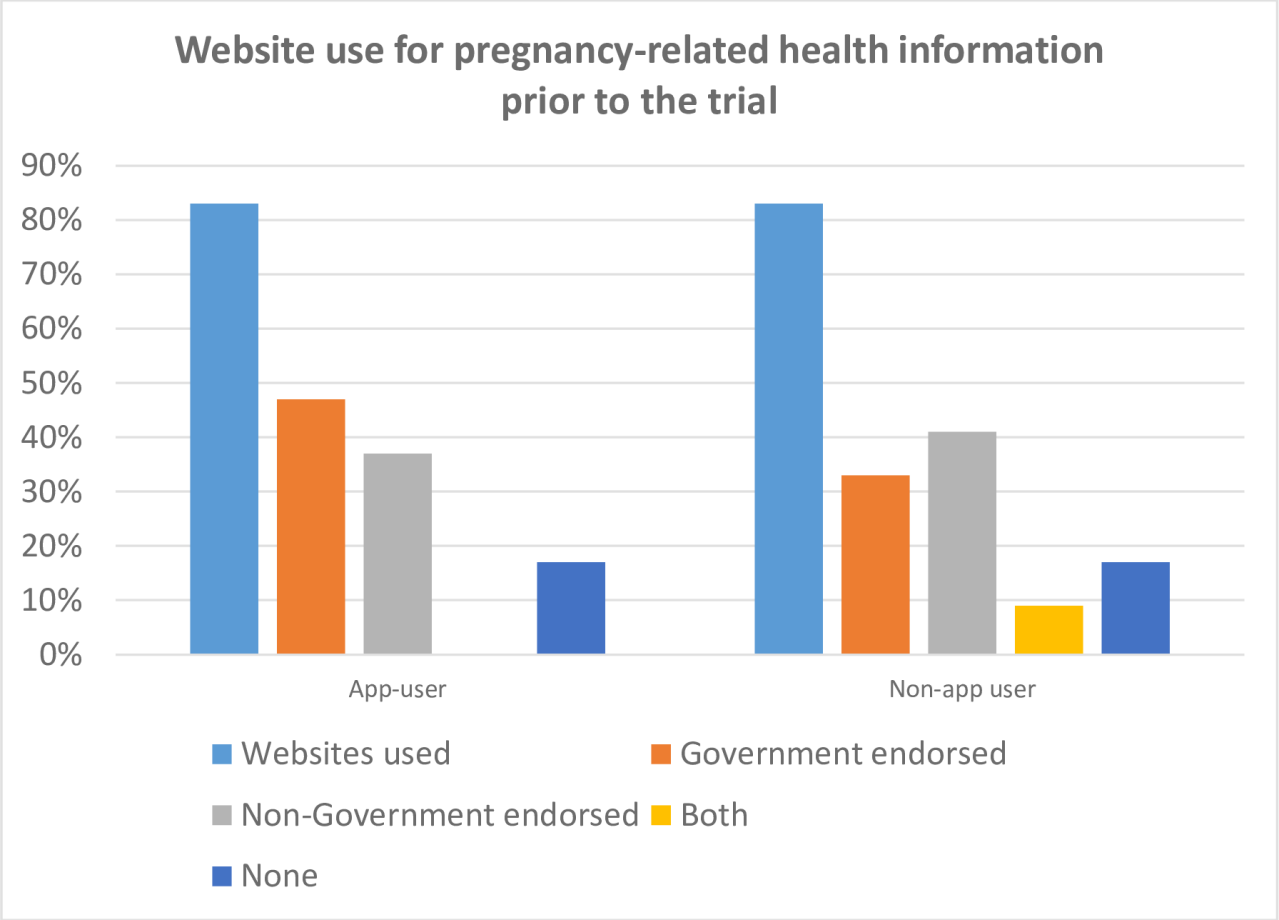
Website use for pregnancy-related health information prior to the trial.

At the time of recruitment participants were asked about their current communication technology use. As indicated in Table 2, 83% of both App user (n = 25) and Non-app user (n = 59) groups had accessed the internet for pregnancy related information prior to their first hospital appointment. There was also no significant difference between the two groups in relation to the websites they accessed (Student t-test, p = 0.311, Fig 3).

**Table 2:** Information and communication technology use for App-user vs Non-App user participants.

	APP USER	NON-APP USER	P value
**PREGNANCY WEBSITES**			.311
Number users (%)	25 (83%)	59 (83%)	
		
Government endorsed	14 (47%)	31 (33%)	
Non-Government	11 (37%)	39 (41%)	
Both Govt & Non-Govt	0 (0%)	8 (9%)	
None	5 (17%)	16 (17%)	
**MOBILE APPS USED-** **Before trial**			.331
Number users (%)	19 (62%)	50 (53%)	
**MOBILE APPS USED-** **At commencement of Trial**			1.000
Number users (%)	15 (50%)	47 (50%)	

**Fig 4:**
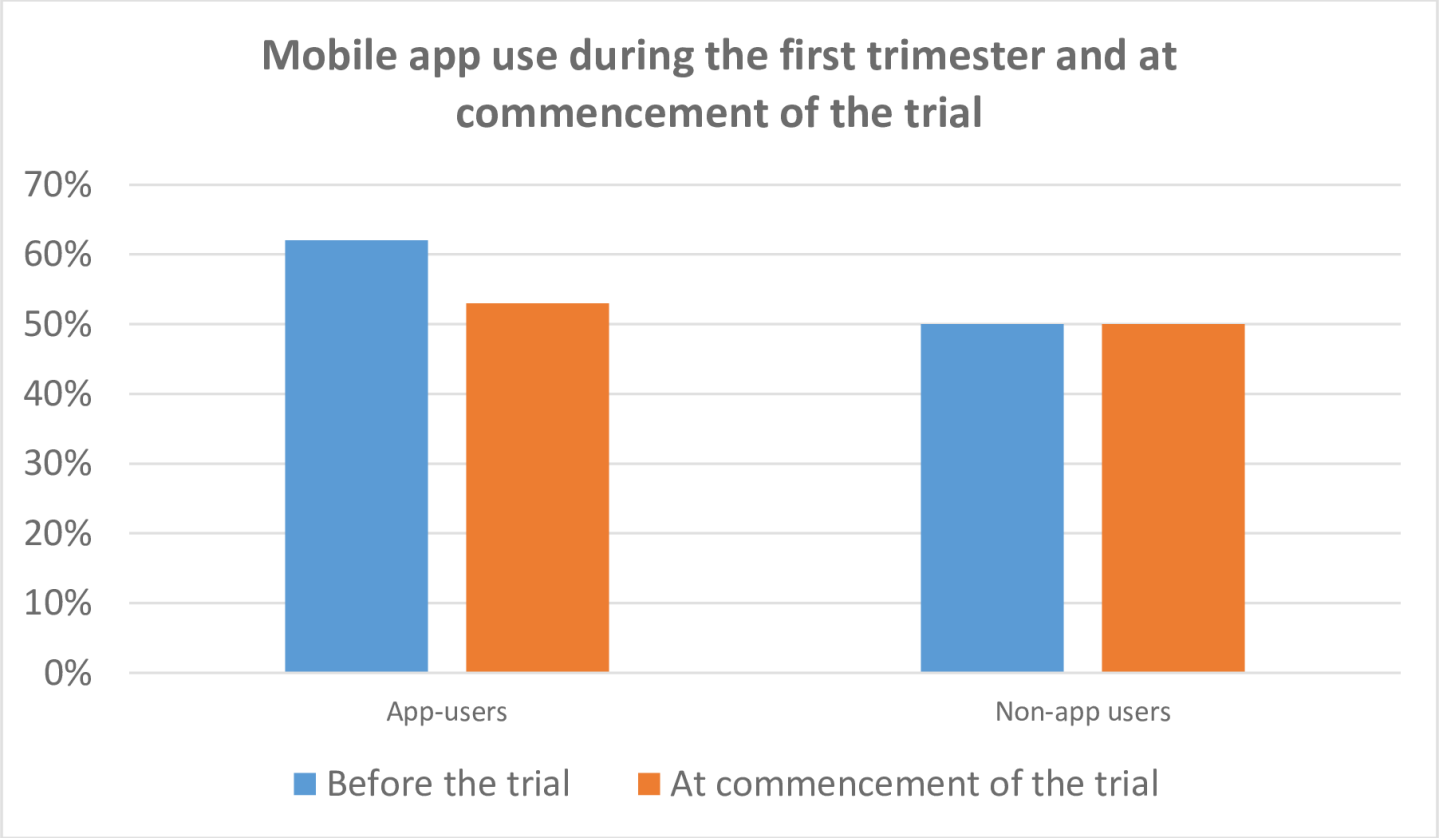
Mobile app use during the first trimester and at commencement of the trial.

Sixty two percent (n = 19) of the App user group had sought other pregnancy apps before the study to obtain pregnancy information, compared to 56% (n = 53) of the Non-app user group. Half of the women in both groups were continuing to use at least one pregnancy app at commencement of the trial (Fig 4).

### Confidence

The participants’ level of confidence in knowing where to access information and help was explored using a self-efficacy scale, self-rating from 0 (no confidence at all) to a rating of 6 (extremely confident). The App users had a confidence score of 4.53 ± 0.23 and Non-App users had a score of 4.6 ± 0.12 and were not significantly different (Student t-test p = 0.73).

### Mental health status

**Fig 5:**
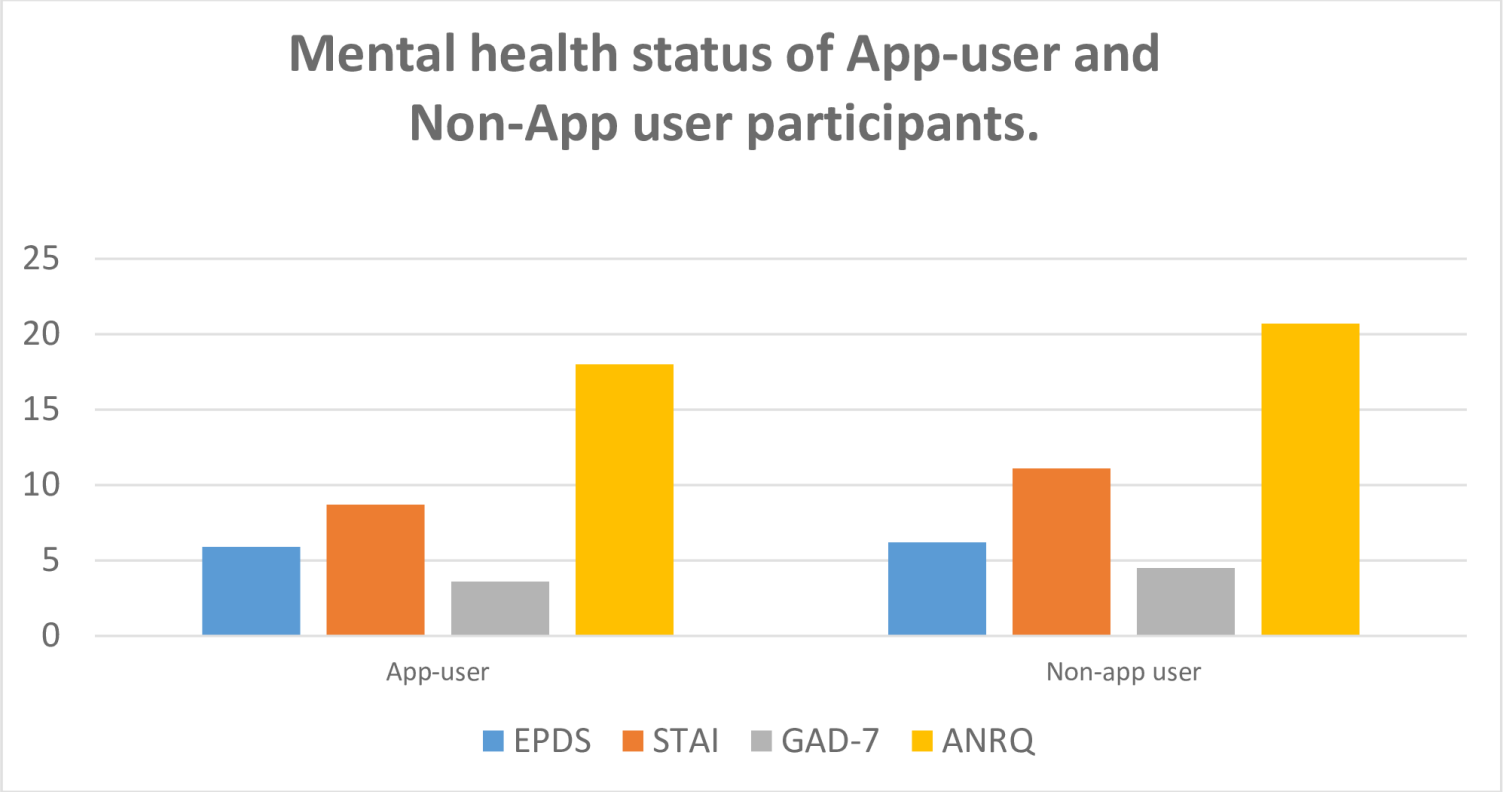
Mental health status of App-user and Non-App user participants.

The assessment of mental health status at the commencement of the trial, showed no significant differences between the App user and Non-app user in the ANRQ, EPDS, GAD-7 scores, however the STAI showed a significant difference (Fig 5). The STAI for the Non-app users reflected a higher level of anxiety (Student t-test, p = 0.001). The mental health status of the App-user group did not change from the commencement and completion of the trial (Table 3)

**Table 3:** Mental health status at commencement and completion of the trial for App-users.

	Commencement	Completion	P value
	**EPDS**	**EPDS 2**	.635
Mean	6.0769	5.6538	
N	26 (87%)	26 (87%)	
Standard Error	1.29487	1.26606	
Range	0–28	0–28	
	**GAD-7**	**GAD-7 2**	.782
Mean	3.6552	3.4828	
Standard Error	0.67553	0.87469	
N	29 (97%)	29 (97%)	
Range	0–12	0–18	
	**STAI**	**STAI 2**	.581
Mean	8.7586	9.1724	
N	29 (97%)	29 (97%)	
Standard Error	0.53373	0.59981	
Range	6–16	6–22	

### Maternal Antenatal Attachment

The Maternal Antenatal Attachment Scale scores for both groups were not statistically different (App users: 75.6 ±1.79 vs Non App users: 76.6 ±1.1).

### Parenting confidence

The Parenting Sense of Competence scores for both groups were not statistically different (App users: 39.4 ±1.9 vs Non App users: 39.9 ±1.1)

## Discussion

Our research prior to and during the development of the Health-e Babies App combined with the analysis of the data from the App-user group demonstrated that use of a mobile phone pregnancy app by a specific group of women derived from a socially disadvantaged community has the potential to inform, educate and change behaviour [1]. However, the high rate in non-completion of the study requirements is concerning. The design of the pilot study and the limitations of the Health-e Babies App itself (i.e. no way to remotely monitor app usage) meant that the researchers were not able to determine if women in the ‘Non-app’ user group used the App, and if so, how, when and why. Nevertheless, from the data we have collected it appears women with a high level of anxiety and financial impairment may have had major barriers to engaging with the mobile phone app. This relationship would be better examined by a randomised control trial in the future. The lack of participation in research has previously been attributed to cultural differences, language barriers, socio-economic status, lower financial constraints and time constraints [29, 30]. Whilst cultural and language barriers should not have been an issue in this group, the other factors may well have contributed to the low rate of completion of the study.

Socially disadvantaged populations have been shown to be less likely to comply with health interventions [31], whether this be due to lack of education, interest or motivation due to poor health literacy [32]. [33] and Krebs [32] reported that those who have limited education are less likely to download a health app than those with tertiary qualifications. A recent characterisation of medical apps [34] indicated poor instruction on how to use an app was a barrier to their uptake. In our study we intentionally left subjects to manage the app on their own for the purpose of defining its useability and this may have been one of the issues affecting the high dropout rate in the study.

Published reports on mobile app research rarely mention the drop-out rate of participants but simply the outcome of data collated by those completing the studies [35, 36]. Numerous studies focus on levels of engagement, interactivity and interest [37] as a means of evaluating the effectiveness of mobile health apps and others gauge the success of apps by their ability to remain in the top of the ‘Top 300 Chart’ of the mobile app stores, the scope of their market and period of time the apps were used by consumers [38]. However, when it comes to health apps, few consider those who would benefit from the use of health apps but choose not to download the app. A national survey on health app use in the United States of America reported the main reasons for non-use of apps include disinterest, cost and the fear of data collection by unknown persons via the app [32]. It was also reported that those more likely to download an app were younger and tertiary educated.

The factor of non-participation is important in relation to health outcomes and community health. The ‘best app’ may show improved health outcomes but if the people who need it the most cannot get it or do not know how to use it, then it is of limited value.

Commercial apps downloaded from app stores such as ‘Google Play’ (Android) and the ‘App Store’ (iOS) are easily downloaded and data can be remotely collected about the purchaser’s use of the app [39]. These app users choose to download the app for their own personal reasons and motivation. There is often no clear recognition that the apps have reached their targeted population [39]. There have been few randomised controlled trials where participants are given the app as an intervention of care [40] and some simply report on the estimate of effectiveness of the app made by those using it [39, 41]. In addition, even when attempting to employ a more sophisticated methodology, studies still tend to report only on the participants who completed the study and not those who failed to complete it [36]. Whilst they may reach part of their target cohort, they are not recognising any gaps within it.

### The effects of socio-economic factors

Differences between socio-economic groups and their adoption of the use of mobile health applications have rarely been reported and studies have not explored ways to address any incongruities [38–40, 42]. [43] identified language, cultural issues, socio-economics and remoteness of community as potential barriers to mobile technology use for Indigenous Australian communities but failed to suggest any specific means to resolve them.

In this study, both the App-user and the Non-app user groups showed no significant difference in socio-economic status except for their employment status. Despite this, the high level of single marital status in the most disadvantaged region in metropolitan Australia, could still play a strong part in the lack of participation. [44] reported a close relationship between single-parent status and anxiety and depression. Single parents, particularly young mothers, often have less contact with family and friends, thus limiting their support network, reducing self-confidence and increasing the likelihood of developing depression [45]. Further to this, women who live in poverty and experience its associated stresses, have more difficulty in developing coping mechanisms [46]. Consequently, this affects their focus on important aspects of their life such as pregnancy [47]. This belief was congruent with a study conducted at the trial hospital which revealed that pregnant women attending this hospital were extremely vulnerable due to a high level of exposure to domestic violence, particularly during pregnancy, and previous physical and emotional abuse during their own childhood [16]. Competing priorities can therefore affect pregnant women’s ability to engage with the app or result in a lack of interest for research. It is therefore argued that addressing barriers such as these are imperative to improve engagement and health education for women living in such circumstances. Strategies to increase engagement based on the findings of this pilot trial are provided below.

Health consciousness (understanding one’s own health status and health needs) has been reported to be an indicator of higher health app use [48] and suggested that the acceptance and use of mobile apps is influenced by an individual’s belief of its usefulness, thoughts of risk of the use and the degree to which they are focussed on their own health [35]. Therefore the value of downloading and use of a pregnancy app may need to be strongly emphasised to the target group, to enable them to understand why it is important and how it would benefit them.

This study has demonstrated that mobile apps are frequently used, with 50% of both groups of women claiming to be using at least one other pregnancy app before the trial. This suggests that apps are a popular medium for receiving pregnancy-related health information. However the additional charges associated with the use of data on a mobile phone or tablet device such as extra data [49] and access to external websites plus an inability to purchase new data immediately when needed may limit app use. Also the more familiar and efficient a person is with using a health app, the less likely they are to replace it with a different health app [48]. Therefore, if women were satisfied with an existing pregnancy app that they used prior to the introduction of the Health-e Babies App, they may have been less interested in downloading and trialling this new app.

There is also evidence from this study that Non-app users have a limited understanding of mobile technology, given that eleven women (7%) reported problems downloading the app. This is in contrast to Daniels and Wedler’s [8] view that all women are technologically savvy. Our study suggests that greater attention needs to be paid to women’s level of technological literacy in order to ensure that apps are accessible. Some women may need further instruction outlining how to download the app successfully and how to use it. Therefore technological literacy must be addressed to ensure participant’s optimal use and confidence in using the app [50]. As with most educational technologies, there is a need for complementary methods to support the app such as social reinforcement by others, rather than assuming that it will work effectively all by itself [51] [52].

### Strategies for engagement

Various strategies have been suggested to promote use and engagement with the pregnancy apps. These include:

1. Downloading the app for the participant at the time of recruitment, to ensure compatibility with their mobile device. However, this may not be practical given the time constraints experienced by antenatal healthcare providers.
2. Showing how the contents of the App are relevant to the individual by explaining the features that would help them with their specific needs and existing media habits.
3. Minimising the need for access to websites externally from the app by having sufficient information on the app itself. This would reduce the financial costs associated with data usage that may have been a factor that shaped women’s app use.
4. Meeting the needs of participants with low literacy by providing them with audio recordings of information that is also given in written format in the app and providing audio-visual displays and diagrams. Where these are features of an app (as in the Health-e Babies App) it is important that researchers ensure participants are aware of these capabilities.
5. Sending a follow-up text message to the participant one week after the initial recruitment consultation to determine if they are having any problems with the app. This will also act as a reminder to look at the app.
6. Embedding notifications and text messages to promote engagement [53–55]. Enable a means of two-way communication via the App between the woman and health professional [11, 56].
7. Educating midwives and doctors about how they can utilise the app at the point of consultation (i.e. using app content as an education tool in antenatal appointments). The use of the app at consultations can provide greater confidence in the app as an informational tool for women, thereby promoting its use [12, 57].
8. Countering the trend of frequent changing of mobile phones by ensuring easy access to the acquisition of the app through an online app store or website optimised for both iOS and Android mobile technology.

### App design

The design of the Health-e Babies App was considered to be good by the App-user group but this could not be determined by the Non-app users. Whether or not the App was appealing to the Non-app user group will never be known nor if this was a factor in their non-participation with the study. Had this group been contactable and willing to respond, interviews could have been conducted to better evaluate the App design.

The design of the App provided audio-recorded relaxation meditations and tips for reducing anxiety but without remote access to data use, it was impossible to determine the effectiveness of this tool. Future research into the effectiveness of mental health tools within the app could be implemented if remote access could be achieved.

## Limitations

A limitation of the study was that women with non-Android mobile phones were excluded due to the app having been developed only for Android smart phones. This resulted in approximately 50% of women who otherwise met the inclusion criteria, were unable to trial the Health-e Babies App. Free wifi was unavailable at the time of recruitment, thus many participants were unable to download the app in the presence of the researcher. This meant that any difficulties downloading the app could not be addressed immediately.

As previously mentioned, the limited capabilities of the app resulted in the inability to identify the Non-app user group member’s actual use of the app during the study period (i.e. analysing app data usage was reliant on downloading material from women’s phones in person). Whilst it might be assumed that women who did not continue in the trial did not use the app, this may not be correct. A randomised control trial would have given more definitive results. The use of telephone interviews and/or face-to-face focus groups would also have been appropriate methods to utilise in order to further examine how pregnant women experienced the app (i.e. perceived effectiveness, everyday routines informing use, likes/dislikes, etc.). However, this was a pilot study that was designed to assist in decision-making for the development of a full pregnancy app. Future research in this area should carefully consider how women’s access and use of pregnancy apps will be measured and what strategies could be implemented if there is a high drop-out rate.

Further to this, women experiencing domestic violence was not explored in the initial questionnaires. This limited the ability to better understand the circumstances that these women were living in, particularly as this community has been previously characterised as having a high level of domestic violence [16]. This variable could have had had an impact on the use of the App and participation in the research. In future this could be explored when evaluating the use of a pregnancy app for socio-economically disadvantaged women.

## Conclusion

Research into mobile app use has predominately focused on how app usage affects participant behaviour, modification of lifestyle, and effectiveness in disease management and prevention [36, 39, 58–60]. However, inaccurate assumptions are often made about people’s ability to access and use mobile technology [10]. This study highlights the importance of understanding the difficulties that women living in socio-economically disadvantaged communities may face and how these challenges can impact their ability to engage with health professionals, educational apps and research projects more broadly. It is imperative that these factors are considered if mobile phone apps are to become a viable medium of health communication for disadvantaged populations.

## Supporting information

S1 File Health-e Babies App Questionnaires- includes Demographic/ ICT Questionnaire, STAI: State Trait Anxiety Inventory, GAD-7: Generalised Anxiety Disorder 7 point anxiety assessment, MAAS: Maternal Antenatal Attachment Score and PCoS: Parenting Sense of Competence. (PDF)

S2 File ANRQ- Antenatal Risk Assessment Questionnaire. (PDF)

S3 File EPDS- Edinburgh Postnatal Depression Score. (PDF)

S4 File 22 Week Questionnaires- includes the Health-e Babies App Questionnaire, STAI: State Trait Anxiety Inventory, GAD-7: Generalised Anxiety Disorder 7 point anxiety assessment, MAAS: Maternal Antenatal Attachment Score and PCoS: Parenting Sense of Competence. (PDF)

S5 File Health-e Babies App copy of data files. (PDF)

## Abbreviations

App: mobile phone application
ICT: Information and Communication Technology
EPDS: Edinburgh Postnatal Depression Score
ANRQ: Antenatal Risk Questionnaire
STAI: State Trait Anxiety Inventory
GAD-7: Generalised Anxiety Disorder 7 point anxiety assessment
MAAS: Maternal Antenatal Attachment Score
PCoS: Parenting Sense of Competence
