# Supplementary material for: The Health-e Babies App for antenatal education: Feasibility for socially disadvantaged women
Source: PLoS One. 2018 May 16;13(5):e0194337. doi: 10.1371/journal.pone.0194337 (PMC5955503; doi:10.1371/journal.pone.0194337)
Supplement: S2 File — (PDF) [file pone.0194337.s002.pdf]

MR 049

Lyell McEwin Hospital

Government of South Australia
SA Health

Antenatal Risk Questionnaire

Patient Identification

UR No: ____________________

Surname: ____________________

Given Names: ____________________

D.O.B. ____________________ Sex ____________

Area/Ward ____________________ OPD ____________

Name ____________________ DOB ____/____/____

Today's Date ____/____/____ Baby Due ____/____/____

Phone (H)____________________ (W)____________________ (M)____________________

ANRQ Total [ ]

This is part of your antenatal booking evaluation and will guide us as to what services we can offer you during your pregnancy. It is confidential information and will remain in your file.

Instructions: Circle numbers 1 - 6 or tick Yes/No as applicable. **Please complete All items**

OFFICE USE

1. When you were growing up, did you feel your mother was emotionally supportive of you? *(If you had no mother circle 6).*

1	2	3	4	5	[6
very much		somewhat		not at all	[

2. a) Have you ever had 2 weeks or more when you felt particularly worried, miserable or depressed? **Yes** ☐ **No** ☐

b) Do you have <u>any other history of mental health problems</u>? **Yes** ☐ **No** ☐
e.g. eating disorders, psychosis, bipolar, schizophrenia. Please Specify: ____________________

If Yes to 2a or 2b, did this:

c) Seriously interfere with your work and your relationships with friends and family?

1	2	3	4	5
not at all		somewhat		very much

d) Lead you to seek professional help? **Yes** ☐ **No** ☐
Did you see a: Psychiatrist ☐ Psychologist/counsellor ☐ GP ☐ ____________________
(Name of professional)

e) Did you take tablets / herbal medicine? No ☐ Yes ☐ Please specify: ____________________

3. Is your relationship with your partner an emotionally supportive one? *(If you have no partner circle 6).*

1	2	3	4	5	[6
very much		somewhat		not at all	[

4. a) Have you had any stresses, changes or losses in the last 12 months? **Yes** ☐ **No** ☐
(eg. separation, domestic violence, job loss, bereavement)
Please list: ____________________

b) How distressed were you by these stresses, changes or losses?

1	2	3	4	5
not at all		somewhat		very much

5. Would you generally consider yourself a worrier?

1	2	3	4	5
not at all		somewhat		very much

6. In general, do you become upset if you do not have order in your life? (eg. regular time table, a tidy house)

1	2	3	4	5
not at all		somewhat		very much

7. Do you feel you have people you can depend on for support with your baby?

1	2	3	4	5
very much		somewhat		not at all

8. Were you emotionally abused when you were growing up? **Yes** ☐ **No** ☐

9. Have you <u>ever</u> been sexually ☐ or physically ☐ abused? **Yes** ☐ **No** ☐

If you would like to seek some help with any of these issues please discuss them with your midwife or doctor.

Would you be happy to be contacted by a health professional during your pregnancy for support and information? **Yes** ☐ **No** ☐

Do you consent to these results being forwarded to your GP? **Yes** ☐ **No** ☐

ANTENATAL RISK QUESTIONNAIRE

MR 049

Catalogue 7530F6074
Created 06/2010

Devised by M-P Austin & SESAHS. Permission given by author to reproduce.
