# Supplementary material for: The Health-e Babies App for antenatal education: Feasibility for socially disadvantaged women
Source: PLoS One. 2018 May 16;13(5):e0194337. doi: 10.1371/journal.pone.0194337 (PMC5955503; doi:10.1371/journal.pone.0194337)
Supplement: S3 File — (PDF) [file pone.0194337.s003.pdf]

MR 050

Lyell McEwin Hospital

Government of South Australia

SA Health

Edinburgh Postnatal Depression Scale

Patient Identification

UR No: ______

Surname: ______

Given Names: ______

D.O.B. ______ Sex ______

As you are pregnant, or have recently had a baby, we would like to know how you are feeling now. Please UNDERLINE the answer which comes closest to how you have felt IN THE PAST WEEK.
Here is an example, already completed.
I have felt happy:

Todays date ______

Yes, all the time
<u>Yes, most of the time</u>
No, not very often
No, not at all

This would mean: 'I have felt happy most of the time' during the past week. Please complete the other questions in the same way.

IN THE PAST 7 DAYS

SCORE

1. I have been able to laugh and see the funny side of things:
- As much as I always could — 0
- Not quite so much now — 1
- Definitely not so much now — 2
- Not at all — 3

2. I have looked forward with enjoyment to things:
- As much as I ever did — 0
- Rather less than I used to — 1
- Definitely less than I used to — 2
- Hardly at all — 3

3. I have blamed my self unnecessarily when things went wrong:
- Yes, most of the time — 3
- Yes, some of the time — 2
- Not very often — 1
- No, never — 0

4. I have felt anxious or worried for no very good reason:
- No, not at all — 0
- Hardly ever — 1
- Yes, sometimes — 2
- Yes, very often — 3

5. I have felt scared or panicky for no very good reason:
- Yes, quite a lot — 3
- Yes, sometimes — 2
- No, not much — 1
- No, not at all — 0

6. Things have been getting on top of me:
- 3 — Yes, most of the time I haven't been able to cope at all
- 2 — Yes, sometimes I haven't been coping as well as usual
- 1 — No, most of the time I have coped quite well
- 0 — No, I have been coping as well as ever

7. I have been so unhappy that I have had difficulty sleeping:
- 3 — Yes, most of the time
- 2 — Yes, sometimes
- 1 — Not very often
- 0 — No, not at all

8. I have felt sad or miserable:
- 3 — Yes, most of the time
- 2 — Yes, quite often
- 1 — Not very often
- 0 — No, not at all

9. I have been so unhappy that I have been crying:
- 3 — Yes, most of the time
- 2 — Yes, quite often
- 1 — Only occasionally
- 0 — No, never

10. The thought of harming myself has occurred to me:
- 3 — Yes, quite often
- 2 — Sometimes
- 1 — Hardly ever
- 0 — Never

Would you be happy to be contacted by a health professional during your pregnancy for support and information? Yes ☐ No ☐

Do you consent to these results being forwarded to your GP? Yes ☐ No ☐

EDINBURGH POSTNATAL DEPRESSION SCALE

MR 050

Catalogue 7530F 6060
Revised 04/2011

(Cox et al 1985)
