# Supplementary material for: The Health-e Babies App for antenatal education: Feasibility for socially disadvantaged women
Source: PLoS One. 2018 May 16;13(5):e0194337. doi: 10.1371/journal.pone.0194337 (PMC5955503; doi:10.1371/journal.pone.0194337)
Supplement: S4 File — (PDF) [file pone.0194337.s004.pdf]

HEALTH-e BABY – SOCIAL MEDIA

QUESTIONNAIRE / INTERVIEW QUESTIONS

ID CODE:

DATE:

1. How often did you look at the Health-e Baby phone app provided to you as part of this study?
   Please tick the most appropriate box.
   - ☐ More than once a day
   - ☐ At least once a day
   - ☐ At least once a week
   - ☐ At least once a fortnight
   - ☐ At least once a month
   - ☐ Less than once a month
   - ☐ Only once when it was installed on my phone

The Appearance of the App

2. Did the Health-e Baby app use appropriate **colours**?
   Please circle your choice on the scale below.

Not at all appropriate			OK			Perfect
1	2	3	4	5	6	7

3. Did the Health-e Baby app use appropriate **icons**?
   Please circle your choice on the scale below

Not at all appropriate			OK			Perfect
1	2	3	4	5	6	7

4. Did the Health-e Baby app use appropriate **pictures**?
   Please circle your choice on the scale below

Not at all appropriate			OK			Perfect
1	2	3	4	5	6	7

5. Did the Health-e Baby app use appropriate **sound** content?
   Please circle your choice on the scale below

Not at all Appropriate			OK			Perfect
1	2	3	4	5	6	7

6. How easy or hard was it to use the Health-e Baby app **functions**?
   Please circle your choice on the scale below.

Impossible to use any features			Variable / some difficulty			Very easy to use all features
1	2	3	4	5	6	7

7. How easy or hard was it to find **information** you needed on the Health-e Baby app?
   Please circle your choice on the scale below.

Impossible to find any information I wanted.			Variable/Some difficulty			Very easy to find all the information I wanted
1	2	3	4	5	6	7

8. How easy or hard was it to understand the **words and phrases** used on the Health-e Baby app?
   Please circle your choice on the scale below.

Couldn't understand Any			Understood Some			Understood All
1	2	3	4	5	6	7

9. How useful was the **information** you received from the Health-e Baby app?
   Please circle you choice on the scale below.

Not at all Useful			Somewhat Useful			Very Useful
1	2	3	4	5	6	7

10. Please complete the following sentence:

The most useful information on the Health-e Baby app was____________, because

____.

11. Was there any information **NOT** included on the Health-e Baby app that would have been helpful? Please list below:

____

____

____

If you would like to participate in a Focus Group to further discuss your opinion of the Health-e Baby app, please leave your name and phone number for Julia (Research Midwife) to call you.

Name: ……………………………………　Phone Number:…………………………………………..

Thank you for your participation in the Health-e Baby Study

MR 050

Lyell McEwin Hospital

Government of South Australia

SA Health

Edinburgh Postnatal Depression Scale

Patient Identification

UR No: ____________________

Surname: ____________________

Given Names: ____________________

D.O.B. ____________________ Sex ____________

As you are pregnant, or have recently had a baby, we would like to know how you are feeling now. Please UNDERLINE the answer ~~which comes~~ closest to how you have felt IN THE PAST WEEK.
Here is an example, already ~~completed~~.
I have felt happy:

Todays date ____________

Yes, all the time
Yes, most of the time
No, not very often
No, not at all

This would mean: 'I have felt happy most of the time' during the past week. Please complete the other questions in the same way.

IN THE PAST 7 DAYS

1. I have been able to laugh and see the funny side of things:
As much as I always could
Not quite so much now
Definitely not so much now
Not at all

2. I have looked forward with enjoyment to things:
As much as I ever did
Rather less than I used to
Definitely less than I used to
Hardly at all

3. I have blamed my self unnecessarily when things went wrong:
Yes, most of the time
Yes, some of the time
Not very often
No, never

4. I have felt anxious or worried for no very good reason:
No, not at all
Hardly ever
Yes, sometimes
Yes, very often

5. I have felt scared or panicky for no very good reason:
Yes, quite a lot
Yes, sometimes
No, not much
No, not at all

6. Things have been getting on top of me:
Yes, most of the time I haven't been able to cope at all
Yes, sometimes I haven't been coping as well as usual
No, most of the time I have coped quite well
No, I have been coping as well as ever

7. I have been so unhappy that I have had difficulty sleeping:
Yes, most of the time
Yes, sometimes
Not very often
No, not at all

8. I have felt sad or miserable:
Yes, most of the time
Yes, quite often
Not very often
No, not at all

9. I have been so unhappy that I have been crying:
Yes, most of the time
Yes, quite often
Only occasionally
No, never

10. The thought of harming myself has occurred to me:
Yes, quite often
Sometimes
Hardly ever
Never

Would you be happy to be contacted by a health professional during your pregnancy for support and information? Yes ☐ No ☐

Do you consent to these results being forwarded to your GP? Yes ☐ No ☐

Revised 04/2011 Catalogue 7530F 6060

EDINBURGH POSTNATAL DEPRESSION SCALE

MR 050

GAD-7 Anxiety

Over the last two weeks, how often have you been bothered by the following problems?	Not at all	Several days	More than half the days	Nearly every day
1. Feeling nervous, anxious, or on edge	0	1	2	3
2. Not being able to sleep or control worrying	0	1	2	3
3. Worrying too much about different things	0	1	2	3
4. Trouble relaxing	0	1	2	3
5. Being so restless that it is hard to sit still	0	1	2	3
6. Becoming easily annoyed or irritable	0	1	2	3
7. Feeling afraid, as if something awful might happen	0	1	2	3

Column totals ____ + ____ + ____ + ____ =

Total score ______

If you checked any problems, how difficult have they made it for you to do your work, take care of things at home, or get along with other people?

Not difficult at all	Somewhat difficult	Very difficult	Extremely difficult
☐	☐	☐	☐

Source: Primary Care Evaluation of Mental Disorders Patient Health Questionnaire (PRIME-MD-PHQ). The PHQ was developed by Drs. Robert L. Spitzer, Janet B.W. Williams, Kurt Kroenke, and colleagues. For research information, contact Dr. Spitzer at ris8@columbia.edu. PRIME-MD® is a trademark of Pfizer Inc. Copyright© 1999 Pfizer Inc. All rights reserved. Reproduced with permission

STATE TRAIT ANXIETY INVENTORY QUESTIONNAIRE

Question	Please mark the appropriate number on the scale			
1. I feel calm	Not at all<br>1	Somewhat<br>2	Moderately<br>3	Very Much<br>4
2. I am tense	Not at all<br>1	Somewhat<br>2	Moderately<br>3	Very Much<br>4
3. I feel upset	Not at all<br>1	Somewhat<br>2	Moderately<br>3	Very Much<br>4
4. I am relaxed	Not at all<br>1	Somewhat<br>2	Moderately<br>3	Very Much<br>4
5. I feel content	Not at all<br>1	Somewhat<br>2	Moderately<br>3	Very Much<br>4
6. I am worried	Not at all<br>1	Somewhat<br>2	Moderately<br>3	Very Much<br>4

THE UNIVERSITY OF ADELAIDE AUSTRALIA

Government of South Australia
SA Health

HEALTH-e BABY STUDY

Maternal Antenatal Attachment Score (MAAS)

ID CODE:

Please tick the box the appropriate box.

1. Over the past two weeks I have thought about, or been preoccupied with the baby inside me
   - ☐ Almost all the time
   - ☐ Very frequently
   - ☐ Frequently
   - ☐ Occasionally
   - ☐ Not at all

2. Over the past two weeks, when I have spoken about or thought about the baby inside me, I got emotional feelings which were:
   - ☐ Very weak or non-existent
   - ☐ Fairly Weak
   - ☐ In between strong and weak
   - ☐ Fairly strong
   - ☐ Very strong

3. Over the past two weeks my feelings about the baby inside me have been:
   - ☐ Very positive
   - ☐ Mainly positive
   - ☐ Mixed positive and negative
   - ☐ Mainly negative
   - ☐ Very negative

4. Over the past two weeks I have had the desire to read about or get information about the developing baby
   - ☐ Very weak or non-existent
   - ☐ Fairly weak
   - ☐ Neither strong nor weak
   - ☐ Moderately strong
   - ☐ Very Strong

5. Over the past two weeks I have been trying to picture in my mind what the developing baby actually looks like in my womb:
   - ☐ Almost all the time
   - ☐ Very frequently
   - ☐ Frequently
   - ☐ Occasionally
   - ☐ Not at all

6. Over the past two weeks I think of the developing baby mostly as:
   - ☐ A real little person with special characteristics
   - ☐ A baby like any other baby
   - ☐ A human being
   - ☐ A living thing
   - ☐ A thing not yet really alive

7. Over the past two weeks I have felt that the baby inside me is dependent on me for it's wellbeing:
   - ☐ Totally
   - ☐ A great deal
   - ☐ Moderately
   - ☐ Slightly
   - ☐ Not at all

8. Over the past two weeks I have found myself talking to my baby when I am alone:
   - ☐ Not at all
   - ☐ Occasionally
   - ☐ Frequently
   - ☐ Very frequently
   - ☐ Almost all the time I am alone

9. Over the past two weeks I think about (or talk to) my baby inside me, my thoughts:
   - ☐ Are always tender and loving
   - ☐ Are mostly tender and loving
   - ☐ Are a mixture of both tenderness and irritation
   - ☐ Contain a fair bit of irritation
   - ☐ Contain a lot of irritation

10. The picture in my mind of what the baby at this stage actually looks like inside of the womb is:
    - ☐ Very clear
    - ☐ Fairly clear
    - ☐ Fairly vague
    - ☐ Very vague
    - ☐ I have no idea at all

11. Over the past two weeks when I think about the baby inside me I get feelings which are:
    - ☐ Very sad
    - ☐ Moderately sad
    - ☐ A mixture of happiness and sadness
    - ☐ Moderately happy
    - ☐ Very happy

12. Some pregnant women sometimes get so irritated by the baby inside them that they feel like they want to hurt or punish it:
    - ☐ I couldn't imagine I would ever feel like this
    - ☐ I could imagine I might sometimes feel like this, but I never actually have
    - ☐ I have felt like this once or twice myself
    - ☐ I have occasionally felt like this myself
    - ☐ I have often felt like this myself

13. Over the past two weeks I have felt:
    - ☐ Very emotionally distant from my baby
    - ☐ Moderately emotionally distant from my baby
    - ☐ Not particularly emotionally close to my baby
    - ☐ Moderately close emotionally to my baby
    - ☐ Very emotionally close to my baby

14. Over the past two weeks I have taken care with what I eat to make sure the baby gets a good diet:
    - ☐ Not at all
    - ☐ Once or twice when I ate
    - ☐ Occasionally when I ate
    - ☐ Quite often when I ate
    - ☐ Every time I ate

15. When I first see my baby after the birth I expect I will feel:
    - ☐ Intense affection
    - ☐ Mostly affection
    - ☐ Dislike about one or two aspects of the baby
    - ☐ Dislike about quite a few aspects of the baby
    - ☐ Mostly dislike

16. When my baby is born I would like to hold the baby:
    - ☐ Immediately
    - ☐ After it has been wrapped in a blanket
    - ☐ After it has been washed
    - ☐ After a few hours for things to settle down
    - ☐ The next day

17. Over the past two weeks I have had dreams about the pregnancy or baby:
    - ☐ Not at all
    - ☐ Occasionally
    - ☐ Frequently
    - ☐ Very frequently
    - ☐ Almost every night

18. Over the past two weeks I have found myself feeling, or rubbing with my hand the outside of my stomach where the baby is:
    - ☐ A lot of times each day
    - ☐ At least once a day
    - ☐ Occasionally
    - ☐ Once only
    - ☐ Not at all

19. If the pregnancy was lost at this time (due to miscarriage or accidental event) without any pain or injury to myself, I expect I would feel:
    - ☐ Very pleased
    - ☐ Moderately pleased
    - ☐ Neutral (Neither sad, nor pleased or Mixed feelings)
    - ☐ Moderately sad
    - ☐ Very sad

THANK YOU FOR COMPLETING THIS QUESTIONNAIRE

Copyright JT Condon (Dept of Psychiarty, Flinders Medical Centre, South Australia)

Parenting Sense of Competence scale

This survey is to help us understand how you feel about being a parent. Whilst we know you are still pregnant and may not be a parent yet, please think about how you feel about this baby and how you feel about being a mother.

Question	Please mark the appropriate number on the scale					
	Strongly agree 1	2	3	4	Strongly disagree 5	6
1. The problems of taking care of a child are easy to solve once you know how your actions affect your child, an understanding I have acquired.	Strongly agree 1	2	3	4	Strongly disagree 5	6
2. Even though being a parent could be rewarding, I am frustrated now while my child is at his/her present age.	Strongly agree 1	2	3	4	Strongly disagree 5	6
3. I go to bed the same way I wake up in the morning, feeling I have not accomplished a whole lot.	Strongly agree 1	2	3	4	Strongly disagree 5	6
4. I do not know why it is, but sometimes when I'm supposed to be in control, I feel more like the one being manipulated.	Strongly agree 1	2	3	4	Strongly disagree 5	6
5. My mother/father was better prepared to be a good mother than I am.	Strongly agree 1	2	3	4	Strongly disagree 5	6
6. I would make a fine model for a new mother to follow in order to learn what she/he would need to know in order to be a good parent.	Strongly agree 1	2	3	4	Strongly disagree 5	6
7. Being a parent is manageable, and any problems are easily solved.	Strongly agree 1	2	3	4	Strongly disagree 5	6
8. A difficult problem in being a parent is not knowing whether you're doing a good job or a bad one.	Strongly agree 1	2	3	4	Strongly disagree 5	6
9. Sometimes I feel like I'm not getting anything done.	Strongly agree 1	2	3	4	Strongly disagree 5	6
10. I meet my own personal expectations for expertise in caring for my child.	Strongly agree 1	2	3	4	Strongly disagree 5	6
11. If anyone can find the answer to what is troubling my child, I am the one.	Strongly agree 1	2	3	4	Strongly disagree 5	6
12. My talents and interests are in other areas, not in being a parent.	Strongly agree 1	2	3	4	Strongly disagree 5	6
13. Considering how long I've been a mother, I feel thoroughly familiar with this role.	Strongly agree 1	2	3	4	Strongly disagree 5	6

14. If being a mother of a child were only more interesting,<br>I would be motivated to do a better job as a parent.	Strongly agree Strongly disagree<br>1 2 3 4 5 6
15. I honestly believe I have all the skills necessary to be a good mother to my child.	Strongly agree Strongly disagree<br>1 2 3 4 5 6
16. Being a parent makes me tense and anxious.	Strongly agree Strongly disagree<br>1 2 3 4 5 6
17. Being a good mother is a reward in itself.	Strongly agree Strongly disagree<br>1 2 3 4 5 6

Thank you for completing this survey.
