# Supplementary material for: The Health-e Babies App for antenatal education: Feasibility for socially disadvantaged women
Source: PLoS One. 2018 May 16;13(5):e0194337. doi: 10.1371/journal.pone.0194337 (PMC5955503; doi:10.1371/journal.pone.0194337)
Supplement: S5 File — (PDF) [file pone.0194337.s005.pdf]

ID	AGE	MARITAL	EDUCATIOI	EMPLOYED	HCC		PHONEPLA	INTERNET	WEBSITES
2	31	Married/D	Certificate	Employed	Yes		Pre-paid	Yes	Govt
3	34	Married/D	Certificate	Employed	No		Phone Plan	Yes	Non-Govt
9	27	Married/D	Year 11	Homedutie	No		Pre-paid	No	None
10	38	Married/D	University	Employed	Yes		Phone Plan	Yes	Non-Govt
14	28	Married/D	Year 12	Employed	No		Phone Plan	Yes	Govt
21	41	Married/D	Certificate	Employed	Yes		Phone Plan	Yes	Govt
24	28	Married/D	Year 12	Employed	No		Phone Plan	Yes	Non-Govt
26	32	Married/D	University	Employed	No		Phone Plan	Yes	Non-Govt
45	34	Married/D	Year 12	Employed	No		Phone Plan	Yes	Non-Govt
55	25	Married/D	Year 11	Employed	No		Phone Plan	Yes	Govt
58	23	Married/D	Year 12	Employed	No		Pre-paid	Yes	Non-Govt
62	25	Married/D	Year 11	Homedutie	No		Pre-paid	No	None
66	26	Married/D	Year 12	Employed	No		Phone Plan	Yes	Non-Govt
70	19	SIngle	Year 11	Employed	Yes		Pre-paid	No	None
80	23	Married/D	Year 10	Homedutie	Yes		Phone Plan	Yes	Govt
85	19	Married/D	Year 12	Homedutie	Yes		Phone Plan	Yes	Govt
97	26	Married/D	University	Employed	No		Phone Plan	Yes	Govt
100	25	SIngle	Year 11	Unemploye	Yes		Phone Plan	Yes	Govt
103	21	Married/D	Year 12	Employed	No		Phone Plan	No	None
107	35	Married/D	Year 12	Employed	No		Phone Plan	Yes	Govt
110	34	Married/D	University	Homedutie	No		Monthly ca	Yes	Non-Govt
113	22	Married/D	Year 12	Employed	No		Phone Plan	No	None
116	23	Married/D	Year 11	Homedutie	Yes		Pre-paid	Yes	Govt
1	22	Married/D	Certificate	Unemploye	No		Phone Plan	Yes	Govt and N
4	20	SIngle	Year 10	Unemploye	Yes		Pre-paid	Yes	Govt
5	20	SIngle	Year 10	Employed	No		Pre-paid	Yes	Non-Govt
6	27	SIngle	Year 10	Homedutie	Yes		Phone Plan	Yes	Govt
8	32	Married/D	Year 12	Employed	No		Pre-paid	No	None
11	30	Married/D	University	Homedutie	Yes		Pre-paid	Yes	Govt
15	27	SIngle	Year 10	Homedutie	Yes		Pre-paid	Yes	Non-Govt
16	21	Married/D	Certificate	Student	Yes		Phone Plan	Yes	Non-Govt
17	29	Separated	Year 12	Employed	Yes		Phone Plan	No	None
18	23	Married/D	University	Employed	No		Phone Plan	Yes	Govt
19	19	Married/D	Year 10	Employed	No		Phone Plan	Yes	Govt
20	24	SIngle	Year 10	Unemploye	Yes		Pre-paid	Yes	Govt
22	32	Married/D	Year 10	Employed	Yes		Pre-paid	Yes	Non-Govt
23	23	SIngle	Year 12	Employed	No		Phone Plan	No	None
25	21	Married/D	Year 10	Employed	No		Phone Plan	Yes	Non-Govt
27	22	SIngle	Certificate	Student	No		Phone Plan	No	None
28	26	SIngle	Year 11	Employed	Yes		Pre-paid	Yes	Non-Govt
29	31	Married/D	Certificate	Homedutie	No		Phone Plan	Yes	Govt
32	25	Married/D	Year 12	Unemploye	Yes		Pre-paid	Yes	Govt
33	23	Married/D	Year 12	Employed	Yes		Pre-paid	No	Govt
34	26	Married/D	University	Employed	No		Monthly ca	Yes	None
36	35	Married/D	Year 11	Homedutie	No		Pre-paid	No	Govt
37	26	Married/D	Year 12	Employed	No		Monthly ca	Yes	None
38	20	SIngle	Year 10	Employed	No		Monthly ca	No	Govt
39	20	Married/D	Year 10	Homedutie	Yes		Pre-paid	Yes	Govt
42	26		Year 12	Employed	No		Phone Plan	Yes	Govt

47	24	Married/D	Certificate	Unemploye	Yes	Pre-paid	No	Non-Govt
48	21	Married/D	Year 11	Homedutie	Yes	Monthly ca	No	None
49	23	Married/D	Year 11	Homedutie	No	Phone Plan	No	None
50	30	Married/D	Certificate	Employed	No	Phone Plan	Yes	None
51	22	Married/D	Year 9	Employed	No	Phone Plan	Yes	Non-Govt
52	18	Married/D	Year 10	Student	Yes	Pre-paid	Yes	Non-Govt
53	27	SIngle	Year 10	Homedutie	Yes	Pre-paid	Yes	Govt
54	33	Married/D	University	Employed	No	Monthly ca	Yes	Govt
57	31	Married/D	Certificate	Employed	No	Monthly ca	Yes	Non-Govt
63	23	Married/D	Certificate	Homedutie	No	Phone Plan	Yes	Govt and N
64	26	SIngle	Year 9	Unemploye	Yes	Pre-paid	No	Non-Govt
65	27	Married/D	University	Homedutie	No	Pre-paid	Yes	None
67	22	Married/D	Year 12	Employed	No	Phone Plan	Yes	Non-Govt
68	27	Married/D	Year 12	Employed	No	Phone Plan	Yes	Govt and N
69	20	Married/D	University	Unemploye	Yes	Phone Plan	Yes	Non-Govt
72	21	SIngle	Year 12	Employed	No	Phone Plan	Yes	Non-Govt
74	23	SIngle	Year 10	Homedutie	Yes	Pre-paid	Yes	Non-Govt
75	23	Married/D	University	Student	No	Phone Plan	No	Govt
77	28	Married/D	University	Homedutie	No	Phone Plan	Yes	Non-Govt
81	24	SIngle	Year 12	Student	Yes	Pre-paid	Yes	Non-Govt
82	32	Married/D	Certificate	Student	Yes	Pre-paid	Yes	Govt and N
83	24	Separated	Year 11	Student	Yes	Pre-paid	No	None
84	26	SIngle	Year 11	Homedutie	Yes	Pre-paid	Yes	Govt
86	22	Married/D	Year 11	Unemploye	No	Pre-paid	Yes	Non-Govt
87	23	SIngle	Certificate	Employed	No	Phone Plan	Yes	Non-Govt
88	29	SIngle	Year 12	Student	Yes	Pre-paid	No	None
89	23	SIngle	Year 11	Employed	No	Phone Plan	Yes	Non-Govt
90	26	Married/D	University	Employed	No	Phone Plan	Yes	Non-Govt
91	32	SIngle	Certificate	Employed	Yes	Phone Plan	Yes	Non-Govt
92	23	Married/D	Year 10	Homedutie	No	Pre-paid	No	None
93	22	Married/D	Certificate	Student	Yes	Pre-paid	Yes	Non-Govt
94	27	Married/D	Year 10	Employed	No	Phone Plan	Yes	Govt
95	20	SIngle	Year 11	Homedutie	Yes	Pre-paid	No	None
98	25	Married/D	Certificate	Employed	No	Phone Plan	Yes	Govt and N
99	18	Married/D	Year 11	Unemploye	Yes	Phone Plan	Yes	Govt
101	27	Married/D	Year 10	Homedutie	Yes	Monthly ca	No	None
102	28	Married/D	Year 11	Employed	No	Phone Plan	Yes	Non-Govt
104	25	Married/D	Year 12	Employed	No	Phone Plan	Yes	Govt and N
105	28	SIngle	Year 12	Homedutie	Yes	Monthly ca	Yes	Govt
106	32	Married/D	Certificate	Homedutie	Yes	Pre-paid	Yes	Non-Govt
107	35	Married/D	Year 12	Employed	No	Phone Plan	Yes	Govt
108	33	Married/D	Year 10	Homedutie	No	Phone Plan	Yes	Govt
109	27	Married/D	Year 12	Employed	Yes	Phone Plan	Yes	Non-Govt
111	29	Married/D	University	Employed	No	Phone Plan	Yes	Govt and N
112	30	Married/D	Certificate	Homedutie	Yes	Pre-paid	Yes	Non-Govt
114	38	Married/D	Certificate	Employed	No	Monthly ca	Yes	Govt
115	36	Married/D	University	Employed	No	Phone Plan	Yes	Govt and N
117	23	Married/D	Certificate	Employed	Yes	Pre-paid	Yes	Non-Govt
118	26	Married/D	Year 11	Unemploye	Yes	Phone Plan	Yes	Non-Govt
119	30	SIngle	Certificate	Homedutie	Yes	Phone Plan	Yes	Non-Govt

120	28	Married/De	Year 11	Employed	Yes	Phone Plan	Yes	Govt
125	26	Married/De	Certificate	Employed	No	Monthly ca	Yes	Non-Govt
126	29	Married/De	Year 12	Employed	No	Phone Plan	Yes	Non-Govt
129	18	Married/De	Year 11	Unemploye	Yes	Phone Plan	No	None
130	27	Married/De	University (	Employed	No	Phone Plan	Yes	Non-Govt
131	17	Married/De	Year 10	Unemploye	Yes	Phone Plan	Yes	Non-Govt
132	21	SIngle	Year 10	Unemploye	Yes	Pre-paid	Yes	Non-Govt
133	27	Married/De	Year 12	Employed	No	Phone Plan	Yes	Govt
134	23	Married/De	Certificate	Employed	Yes	Phone Plan	Yes	Govt
135	19	Married/De	Year 10	Employed	No	Pre-paid	Yes	Govt
136	23	SIngle	Year 12	Employed	No	Phone Plan	Yes	Govt
137	27	Married/De	Certificate	Employed	No	Phone Plan	Yes	Non-Govt
138	22	Married/De	Year 12	Employed	No	Phone Plan	Yes	Non-Govt
139	24	Married/De	Year 11	Unemploye	No	Phone Plan	Yes	Non-Govt
140	21	Married/De	Year 9	Student	Yes	Pre-paid	Yes	Govt
141	29	Married/De	Year 12	Unemploye	No	Phone Plan	Yes	Non-Govt
142	21	Married/De	Year 12	Employed	No	Phone Plan	Yes	Govt
143	28	Married/De	Year 12	Employed	No	Phone Plan	Yes	Govt
144	41	Married/De	University (	Employed	No	Phone Plan	Yes	Govt
145	25	Married/De	Year 12	Employed	No	Phone Plan	Yes	Non-Govt
146	27	Married/De	Year 12	Employed	No	Phone Plan	Yes	Govt
147	26	Married/De	Year 12	Employed	No	Phone Plan	Yes	Non-Govt
148	30	Divorced	Year 10	Employed	No	Monthly ca	Yes	Govt
149	21	Married/De	Year 11	Employed	No	Phone Plan	Yes	Govt
150	27	Married/De	Year 12	Employed	No	Phone Plan	Yes	Non-Govt

APPS	APPSNOW	REMINDER	CONFIDEN(	ETHNICITY	EPDS	GAD7	STAI	MAAS
No	No	Yes	3	Caucasian /	14	4	11	62
No	No	Yes	6	Filipino	0	0	6	90
Yes	Yes	Yes	6	Caucasian /	1	1	6	87
Yes	Yes	Yes	6	Caucasian U	4	1	10	83
Yes	Yes	Yes	4	Caucasian /	3	2	8	70
No	No	Yes	3	Filipino		11	10	88
Yes	Yes	Yes	4	Caucasian /	0	0	6	79
No	No	Yes	4	Vietnamese	7	5	8	61
Yes	Yes	No	6	Caucasian /	6	1	7	84
No	No	Yes	3	Caucasian Australian		2	12	78
No	No	Yes	3	Caucasian /	3	1	7	87
Yes	No	Yes	6	Caucasian /	1	6	6	81
Yes	Yes	Yes	5	Caucasian /	3	1	6	81
Yes	Yes	Yes	6	Caucasian /	11	1	8	68
No	No	Yes	6	Caucasian /	0	4	8	64
Yes	Yes	Yes	5	Caucasian /	0	1	6	84
Yes	No	Yes	2	Caucasian /	2	1	7	72
Yes	Yes	Yes	6	Caucasian /	4	0	7	81
Yes	Yes	Yes	3	Caucasian /	4	4	9	82
Yes	Yes	Yes	6	Caucasian I	15	8	12	79
No	No	Yes	5	Indian	28	12	11	74
Yes	No	Yes	5	Caucasian /	0	0	10	69
Yes	Yes	Yes	5	Caucasian Australian		3	14	79
No	No	Yes	5	Caucasian /	3	1	7	72
No	No	Yes	3	Indian	2	0	6	80
Yes	Yes	Yes	6	Caucasian /	12	10	12	82
No	No	Yes	5	Caucasian /	10	7	12	70
No	No	Unsure	4	Filipino	5	0	9	66
No	No	Yes	6	Caucasian Australian		1	6	80
No	No	Yes	6	Caucasian /	10	7	11	81
Yes	Yes	Yes	6	Caucasian /	7	1	8	84
No	No	Yes	3	Vietnamese	1	0	15	74
Yes	Yes	Yes	4	Caucasian U	0	2	8	82
Yes	Yes	Yes	5	Caucasian /	0	2	9	75
Yes	Yes	Yes	6	Caucasian U	4	8	13	79
Yes	Yes	Yes	4	Caucasian /	26	21	22	66
No	No	Yes	4	Aboriginal	1	7	7	83
No	No	Yes	4	Caucasian Australian		0	6	92
No	No	Yes	3	Other	0	1	6	
No	No	Yes	6	Caucasian /	3	1	10	82
No	No	Yes	3	Caucasian Australian		10	18	70
No	No	Yes	5	Caucasian /	10	7	8	81
No	No	Yes	3	Caucasian /	1	5	11	70
Yes	Yes	No	5	Indian		2	14	71
Yes	Yes	Yes	6	Caucasian /	2	0	6	84
Yes	Yes	Yes	3	Caucasian /	3	2	7	84
Yes	Yes	Yes	5	Caucasian /	11	11	15	67
No	No	Unsure	5	Caucasian /	15	14	16	84
No	No	Yes	5	Caucasian /	2	5	14	66

No	No	Yes	2	Other	18	1	7	72
No	No	Unsure	5	Caucasian Australian		0	9	68
No	No	Yes	4	Caucasian /	2	1	10	69
Yes	Yes	Yes	3	Caucasian /	5	2	12	83
No	No	Yes	4	Caucasian /	14	10	18	77
Yes	Yes	Yes	4	Caucasian |	7	8	14	84
No	No	Yes	3	Caucasian UK		7	17	78
Yes	Yes	Yes	4	Other	8	4	11	80
No	No	Yes	6	Other	0	0	6	88
No	No	Yes	3	Caucasian /	4	3	9	76
No	No	Yes	3	Caucasian /	9	8	15	78
No	No	Yes	4	Other	7	2	12	67
Yes	Yes	Yes	3	Caucasian /	5	4	9	82
Yes	Yes	Yes	6	Caucasian /	3	0	8	78
Yes	Yes	Yes	6	Caucasian /	3	0	7	67
Yes	Yes	Yes	4	Caucasian /	4	8	15	68
No	No	Yes	6	Caucasian /	7	11	14	74
No	No	Yes	3	Caucasian /	5	4	14	58
No	No	Yes	4	Other	3	1	8	89
Yes	No	Yes	5	Other	14	15	10	91
Yes	Yes	Yes	2	Other	8	6	13	85
No	No	Yes	4	Caucasian /	9	6	11	74
Yes	Yes	Yes	6	Caucasian /	9	5	13	72
No	No	Yes	1	Caucasian /	0	4	15	75
Yes	Yes	Yes	4	Caucasian /	2	2	11	75
No	No	Yes	4	Caucasian /	7	2	11	83
No	No	Unsure	5	Caucasian /	2	4	13	72
No	No	Yes	5	Filipino	5	0	13	85
Yes	Yes	Yes	5	Caucasian /	6	5	12	71
Yes	No	Yes	6	Caucasian /	7	3	9	11
No	No	Yes	3	Caucasian /	6	3	12	76
Yes	Yes	Yes	4	Caucasian /	12	7	15	77
Yes	Yes	No	3	Caucasian /	1	0	9	72
Yes	Yes	Yes	6	Caucasian /	2	0	9	78
Yes	Yes	Yes	5	Caucasian /	0	0	15	78
Yes	Yes	Yes	6	Caucasian /	7	4	9	75
No	No	Yes	6	Caucasian /	8	9	11	86
Yes	Yes	Yes	6	Caucasian /	7	7	15	87
Yes	Yes	Yes	6	Caucasian /	4	1	7	86
Yes	Yes	Yes	6	Caucasian /	7	7	15	80
Yes	Yes	Yes	6	Caucasian |	15	8	12	75
No	No	Yes	5	Caucasian /	6	5	10	79
No	No	Yes	6	Caucasian Australian		3	11	81
Yes	Yes	Yes	4	Indian	3	1	11	73
No	No	No	4	Caucasian /	11	6	14	76
Yes	Yes	Yes	5	Caucasian /	5	5	6	74
No	No	Unsure	5	Caucasian UK		2	10	70
Yes	Yes	Yes	6	Caucasian Australian		7	13	76
Yes	Yes	Yes	6	Caucasian Australian		5	12	88
Yes	Yes	Yes	6	Caucasian Australian		15	16	79

Yes	Yes	Yes	6	Caucasian Australian				75
No	No	Yes	4	Caucasian A	5	4	10	67
Yes	Yes	Yes	4	Other	9	3	13	82
No	No	Yes	3	Caucasian Australian		5	13	39
Yes	Yes	Yes	4	Caucasian A	2	3	10	51
No	No	Yes	4	Caucasian A	26	13	16	
Yes	Yes	No	4	Caucasian Australian		1	9	83
Yes	No	Yes	6	Caucasian A	11	6	11	68
Yes	Yes	Yes	4	Caucasian Australian		16	19	81
Yes	Yes	Yes	5	Caucasian A	9	10	10	61
No	No	Yes	3	Caucasian A	16	11	16	89
Yes	Yes	Yes	6	Caucasian A	6	2	14	89
Yes	No	Yes	6	Caucasian A	1	0	8	71
Yes	Yes	Yes	6	Caucasian Australian		6	13	84
Yes	Yes	Yes	3	Caucasian A	8	5	8	74
Yes	Yes	Yes	6	Caucasian A	0	0	6	90
Yes	Yes	Yes	5	Caucasian A	0	0	6	75
Yes	Yes	Yes	6	Caucasian Australian		1	7	77
No	No	Yes	6	Caucasian A	6	6	13	71
No	No	Yes	3	Caucasian A	10	7	9	72
Yes	Yes	Yes	4	Caucasian A	8	1	8	78
Yes	Yes	Yes	5	Caucasian A	6	1	6	92
No	No	Yes	6	Caucasian A	8	5	10	90
No	No	Yes	3	Caucasian A	8	1	6	73
Yes	Yes	Yes	3	Caucasian A	4	4	8	76

MAAStol	MAASacc	MAASple	MAAScom	ANRQ	EPDS2	GAD72	STAI2	MAAS2
8	11	27	16	31	7	1	11	90
14	13	37	25	8	11	7	11	89
14	14	34	25	7	2	2	6	100
12	15	34	22	18	1	0	13	78
12	13	29	18	14				
13	15	35	25		11	9	11	84
12	14	31	22	6	0	0	6	83
7	12	28	14	23	4	7	9	86
12	15	34	23		1	0	8	84
12	13	31	22		0	2	11	82
12	14	36	25	7	3	2	7	81
13	14	32	22	29	2	1	7	82
7	15	35	24	9	5	0	8	78
7	13	31	16	27	2	2	8	75
9	10	28	16	11	0	2	7	75
12	14	33	25	6	2	3	9	85
7	14	31	20	9	0	0	6	85
12	13	33	23	10	4	0	6	86
13	14	34	21	8	7	3	7	89
12	14	33	20	40	5	1	7	78
7	12	28	20		28	18	14	81
7	12	33	19	6	2	2	6	74
14	14	33	23		2	0	8	82
9	14	29	19	18				
7	15	35	23	11				
15	14	32	21	19				
9	14	28	19	28				
7	13	28	18	8				
10	14	34	22					
11	14	33	23	37				
13	15	32	24	21				
11	15	31	17	12				
14	12	33	23	8				
12	11	38	24	8				
10	14	34	21	25				
11	11	25	19	58				
15	12	32	24	30				
15	14	39	25					
			18	16				
11	14	34	23	20				
11	13	27	19					
12	13	35	21	27				
11	14	30	15	12				
10	12	26	23					
10	15	31	20	11				
15	14	35	20	20				
10	13	27	17	16				
10	14	36	24	52				
9	11	27	19	10				

9	14	29	19	42
10	12	29	17	
9	13	25	17	9
13	14	32	22	28
11	14	31	21	33
11	13	36	24	34
12	13	30	23	
11	14	32	23	11
12	15	36	25	5
6	13	34	23	25
11	12	33	22	33
10	13	30	19	8
11	14	34	23	13
10	13	27	18	18
10	11	28	18	8
9	11	28	20	9
14	10	28	23	30
6	12	26	14	43
13	14	39	23	14
13	15	38	25	26
10	15	35	25	11
11	13	34	21	15
9	12	31	20	36
11	11	32	21	28
9	13	33	20	10
13	14	32	24	16
8	14	31	19	10
15	14	32	24	8
9	13	30	19	25
11	13	32	20	16
12	11	33	21	16
9	14	28	21	16
7	14	35	22	11
11	14	32	21	6
14	11	28	21	7
15	14	36	21	33
13	14	35	25	29
12	15	35	24	32
10	14	32	24	29
9	14	32	20	27
12	14	33	20	40
7	15	35	24	13
7	13	29	24	
10	13	31	22	7
9	13	30	22	40
9	13	31	17	20
12	14	29	21	
14	14	37	23	
14	12	29	24	
10	12	31	22	

19	9	6	11	84
8				
17	0	0	7	89
43	5	2	11	85
14	11	6	12	86
48	20	18	21	77
9				
6				
33				
7				
47				
33				
23				
6				
24				
14	9	4	10	82
28	7	3	8	83

MAAStol2	MAASacc2	MAASple2	MAAScomp	PSoCS1	PSoCS2	COMPLIAN	APPuse	Colours
14	14	37	25		48	Compliant	At least on	OK
13	15	36	25	34	24	Compliant	At least on	6
151	15	35	25	35	24	Compliant	Less than o	Perfect
10	14	34	20	27	25	Compliant	At least on	OK
						Compliant	At least on	OK
13	14	34	23	45	54	Compliant	At least on	5
12	14	34	23	29	24	Compliant	At least on	6
15	14	35	22	36	36	Compliant	At least on	5
12	15	30	22	39	31	Compliant	At least on	5
9	14	35	24	55	49	Compliant	At least on	OK
15	14	35	22	38	31	Compliant	At least on	6
12	14	33	23	23	28	Compliant	At least on	Perfect
9	13	35	21	31	37	Compliant	Less than o	5
12	14	33	16	35	30	Compliant	More than	5
10	13	31	21	26	24	Compliant	At least on	OK
13	14	34	24	33	25	Compliant	At least on	5
13	15	35	22	41	28	Compliant	Less than o	5
14	14	34	24	22	22	Compliant	At least on	5
15	14	35	25	35	38	Compliant	At least on	6
				57	43	Compliant	At least on	Perfect
13	12	34	22	54		Compliant	At least on	5
8	13	33	20	35	26	Compliant	At least on	5
14	14	34	20	53	41	Compliant	More than	6
				35		Non-Compliant		
				20		Non-Compliant		
				40		Non-Compliant		
				43		Non-Compliant		
				49		Non-Compliant		
				34		Non-Compliant		
				23		Non-Compliant		
				19		Non-Compliant		
				46		Non-Compliant		
				30		Non-Compliant		
						Non-Compliant		
				37		Non-Compliant		
				56		Non-Compliant		
				47		Non-Compliant		
				36		Non-Compliant		
				45		Non-Compliant		
				30		Non-Compliant		
				61		Non-Compliant		
				40		Non-Compliant		
				29		Non-Compliant		
				50		Non-Compliant		
				34		Non-Compliant		
				25		Non-Compliant		
				42		Non-Compliant		
				40		Non-Compliant		
				55		Non-Compliant		

35	Non-Compliant
30	Non-Compliant
42	Non-Compliant
45	Non-Compliant
45	Non-Compliant
34	Non-Compliant
30	Non-Compliant
41	Non-Compliant
17	Non-Compliant
25	Non-Compliant
58	Non-Compliant
32	Non-Compliant
39	Non-Compliant
36	Non-Compliant
29	Non-Compliant
49	Non-Compliant
53	Non-Compliant
51	Non-Compliant
52	Non-Compliant
43	Non-Compliant
30	Non-Compliant
36	Non-Compliant
52	Non-Compliant
42	Non-Compliant
41	Non-Compliant
51	Non-Compliant
31	Non-Compliant
37	Non-Compliant
48	Non-Compliant
42	Non-Compliant
48	Non-Compliant
36	Non-Compliant
36	Non-Compliant
34	Non-Compliant
	Non-Compliant
26	Non-Compliant
43	Non-Compliant
33	Non-Compliant
42	Non-Compliant
44	Non-Compliant
57	Non-Compliant
31	Non-Compliant
42	Non-Compliant
59	Non-Compliant
56	Non-Compliant
42	Non-Compliant
47	Non-Compliant
35	Non-Compliant
55	Non-Compliant
42	Non-Compliant

		Non-Compliant			
44	43	Compliant	At least on		6
		Non-Compliant			
39		Non-Compliant			
51	41	Compliant	At least on		6
		Non-Compliant			
39		Non-Compliant			
39	44	Compliant	At least on	OK	
53		Non-Compliant			
53	42	Compliant	At least on	OK	
39	51	Compliant	Only once		5
		Non-Compliant			
43		Non-Compliant			
37		Non-Compliant			
48		Non-Compliant			
30		Non-Compliant			
42		Non-Compliant			
30		Non-Compliant			
61		Non-Compliant			
36		Non-Compliant			
43		Non-Compliant			
17		Non-Compliant			
22		Non-Compliant			
43	44	Compliant	At least on		3
52	45	Compliant	At least on	OK	

Icons	Pictures	Sound	Functions	Informatio	Words	Usefulness	CONFIDEN	PARITY
OK	5	OK	6	6	6	6		Nullipara
6	Perfect	6	Very easy t	5	Understoo	6		Nullipara
Perfect	Perfect	Perfect	Very easy t	5	Understoo	Very useful		Multipara
5	5	5	6	5	6	Somewhat useful		Nullipara
OK	OK	OK	5	Some diffic	5	3		Nullipara
5	5	5	6	5	5	6		Nullipara
6	6		Very easy t	6	6	6		Multipara
5	5	5	5	5	5	5		Multipara
5	5	5	6	6	Understoo	5		Multipara
5	5	OK	Very easy t	5	5	5		Nullipara
6	6	6	Very easy t	Very easy t	Understoo	Very useful		Multipara
Perfect	Perfect	Perfect	Very easy t	6	Understoo	6		Multipara
OK	5	OK	6	5	5	5		Multipara
OK	5	OK	6	5	5	5		Nullipara
OK	OK	OK	6	6	6	5		Multipara
3	6	2	5	Very easy t	Understoo	5		Nullipara
5	5	5	5	Some diffic	5	Somewhat useful		Nullipara
5	6	5	Very easy t	6	Understoo	6	Very confi	Multipara
6	5	6	6	6	Understoo	5	Very confi	Multipara
OK	OK	6	6	5	Understoo	Somewhat	Very confi	Nullipara
6	6	6	6	Very easy t	Understoo	Very useful	6	Multipara
6	6	6	Very easy t	Very easy t	Understoo	Very useful	Very confi	Multipara
6	6	OK	6	Very easy t	Understoo	Very useful	Very confi	Multipara
								Nullipara
								Multipara
								Multipara
								Nullipara
								Nullipara
								Multipara
								Multipara
								Multipara
								Nullipara
								Nullipara
								Nullipara
								Multipara
								Nullipara
								Multipara
								Nullipara
								Nullipara
								Nullipara
								Multipara
								Multipara
								Nullipara
								Nullipara
								Nullipara
								Multipara
								Nullipara
								Nullipara
								Nullipara

Nullipara
Nullipara
Nullipara
Nullipara
Nullipara
Nullipara
Nullipara
Nullipara
Nullipara
Nullipara
Nullipara
Nullipara
Nullipara
Nullipara
Nullipara
Nullipara
Nullipara
Nullipara
Nullipara
Nullipara
Nullipara
Nullipara
Nullipara
Nullipara
Nullipara
Nullipara
Nullipara
Nullipara
Nullipara
Nullipara
Nullipara
Nullipara
Nullipara
Nullipara
Nullipara
Nullipara
Nullipara
Nullipara
Nullipara
Nullipara
Nullipara
Nullipara
Nullipara
Nullipara
Nullipara
Nullipara
Nullipara
Nullipara
Nullipara
Nullipara

								Nullipara
6	Perfect	5	Very easy t	5	Understoo	Very useful		Nullipara
								Nullipara
								Nullipara
5	6	5	5	5	6	6	Somewhat	Nullipara
								Nullipara
								Nullipara
OK	OK	OK	6	5	Understoo	5		Nullipara
								Nullipara
3	OK	2	5	5	6	Somewhat useful		Nullipara
OK	5	OK	Some diffic	5	Understoo	3		Nullipara
								Nullipara
								Nullipara
								Nullipara
								Nullipara
								Nullipara
								Nullipara
								Nullipara
								Nullipara
								Nullipara
								Nullipara
								Nullipara
								Nullipara
OK	5	OK	5	5	Understoo	5		Nullipara
5	OK		Some diffic	6	Understoo	5	6	Nullipara
